# Site Agnostic Approach to Early Detection of Cyberbullying on Social Media Networks

**DOI:** 10.3390/s23104788

**Published:** 2023-05-16

**Authors:** Manuel López-Vizcaíno, Francisco J. Nóvoa, Thierry Artieres, Fidel Cacheda

**Affiliations:** 1 CITIC Research Center, Computer Science and Information Technologies Department, Campus de Elviña, 15071 A Coruña, Spain; francisco.javier.novoa@udc.es (F.J.N.); fidel.cacheda@udc.es (F.C.); 2 Aix Marseille University, Université de Toulon, CNRS, LIS, Ecole Centrale Marseille, 13397 Marseille, France; thierry.artieres@lis-lab.fr

**Keywords:** cyberbullying, social networks, early detection, machine learning, text features, multiple-instance learning

## Abstract

The rise in the use of social media networks has increased the prevalence of cyberbullying, and time is paramount to reduce the negative effects that derive from those behaviours on any social media platform. This paper aims to study the early detection problem from a general perspective by carrying out experiments over two independent datasets (Instagram and Vine), exclusively using users’ comments. We used textual information from comments over baseline early detection models (fixed, threshold, and dual models) to apply three different methods of improving early detection. First, we evaluated the performance of Doc2Vec features. Finally, we also presented multiple instance learning (MIL) on early detection models and we assessed its performance. We applied timeawareprecision (TaP) as an early detection metric to asses the performance of the presented methods. We conclude that the inclusion of Doc2Vec features improves the performance of baseline early detection models by up to 79.6%. Moreover, multiple instance learning shows an important positive effect for the Vine dataset, where smaller post sizes and less use of the English language are present, with a further improvement of up to 13%, but no significant enhancement is shown for the Instagram dataset.

## 1. Introduction

The use of social networks has been continuously increasing, with millions of people communicating through them every day. Although social networks are powerful tools that have a positive effect on many users, they also have the potential to negatively affect many others. Smith et al. in [1] define cyberbullying as “an aggressive, intentional act carried out by a group or individual using electronic forms of contact, repeatedly or over time against a victim that cannot easily defend him or herself”, which has a special impact among teenagers due to their mistaken threat perception [2,3].

The negative effects of cyberbullying can go from lower school performance, absenteeism, truancy, dropping out, and violent behaviour [4] to potentially devastating psychological effects such as depression, low self-esteem, suicide ideation, and even suicide [5]. Therefore, the early detection of cyberbullying in social networks has become a critical task to mitigate and reduce the negative effects on its victims [6]. Moreover, the frequency and high propagation rate allowed by technology makes it crucial to identify a cyberbullying scenario to stop the aggression and support the victims, as well as to identify the aggressors.

The main aim of this work is to improve the early detection of cyberbullying on social networks, focusing only on users’ comments, in order to provide a general approach that could be potentially applied to any social media. As presented in [6], the use of comments and user features has been explored, but considering the results obtained as baseline, and to perform a site agnostic approach to the early detection problem, no user features have been used.

We consider the results previously obtained in [6] using the fixed, threshold, and dual early detection models and standard syntactic and semantic features (i.e., term frequency-inverse document frequency and latent Dirichlet allocation) for cyberbullying as baselines, and we explore several alternatives to improve the outcome. For this purpose, we extend our experiments to two different datasets, and the features extracted are limited to the comments (without considering user or media features). Moreover, we propose a new set of features (i.e., Doc2Vec) and we provide two improvements to the baseline models.

In particular, we identify and address the following research questions:

- RQ1: Can Doc2Vec features significantly improve performance for the early detection models?
- RQ2: Can multiple instance learning improve results for the early detection models?

Our contributions can be summarised as follows. (1) We study the cyberbullying early detection problem on social media, from a generic perspective focusing exclusively on users’ comments. (2) We analyse the positive impact of Doc2Vec features on early detection models. (3) We introduce multiple instance learning (MIL) to the early detection models and evaluate the performance improvements.

## 2. Related Work

The detection of cyberbullying using machine learning algorithms has attracted considerable attention from researchers in the last few years, with a growing need for the automatic detection of cyberbullying, especially on social media.

A few interesting works in this field include, for example, some authors’ work on the detection of cyberbullying using textual, audio, and visual features. Their results suggest that audiovisual features can help improve the performance of purely textual cyberbullying detectors [7]. Dinakar et al. [8] use term frequency-inverse document frequency in combination with the identification of profane words to detect cyberbullying on YouTube comments. Zhong et al. [9] combine bag of words (BoW) features with Word2Vec to detect cyberbullying on Instagram. They also include images and caption features, concluding that they can serve as a powerful predictor for future cyberbullying. Other authors use support vector machines (SVM) and a lexical syntactical feature to predict offensive language, achieving high precision values [10]. Also interesting is the work presented in [11], which focuses on network-based features and shows how they can be very helpful for the detection of aggressive user behaviour.

Rafiq et al. provide a dataset for the Vine social network and explore the detection of cyberbullying using several machine learning models, achieving the best results with AdaBoost, closely followed by random forest [12,13]. Following this work, Hosseinmardi et al. [14] used an Instagram dataset to further explore cyberbullying detection by incorporating multimodal text and image features as well as media session data. The results obtained by SVM outperformed other classifiers.

Other common features usually considered for the detection of cyberbullying are profanity [15,16,17,18] or sentiment analysis [19,20,21,22,23,24].

Arif in [25] and Singh et al. in [26] recently provided extensive reviews of relevant research works using machine learning techniques to detect cyberbullying on social media. From a generic perspective, most works use content-based features (such as Tf-idf or Word2Vec) and sentiment analysis as a basis, while others also incorporate different features extracted from the user, such as social features (e.g., followers) or their profile (e.g., age or gender).

Interestingly, although some works consider time features relevant for cyberbullying detection [27,28,29,30], to the best of our knowledge, very little effort is devoted to the early detection of cyberbullying. In fact, most works focus on improving performance regarding how successfully a cyberbullying post or session is identified, and therefore, standard evaluation metrics are used, such as accuracy, precision, recall, F-measure, or area under the curve [31,32,33].

Among those works that provide a time-aware evaluation, we find [34], where the authors focus on an early detection of cyberbullying to minimise the damage caused to victims. A basic time-aware evaluation is defined by dividing the original dataset in 10 independent subsets (or chunks), which are sequentially processed by the models. Performance is measured using F1-score at each chunk, but models are not penalised for late detections, and best scores are obtained mainly after processing at least half the dataset.

One of the research questions in [35] refers explicitly to the capacity of the proposed method, named HENIN, to provide early detection of cyberbullying. However, the authors perform a basic time-aware evaluation using precision and accuracy at *k*, and no penalty is introduced for late detection. Although the authors in [36] claim to be working on the early detection of cyberbullying, they in fact focus on computer network cyberattacks, and the time-aware performance evaluation is just based on the time required to make the prediction. Also in [37], an evaluation based on precision, recall, and F1 is used for the early detection of stress and depression.

Finally, this work is mostly related to our previous work [38], where we study the early detection of cyberbullying on the Vine social network considering owner, media session, video and comment features. The threshold and dual early detection models, which constitute our baselines, are proposed, and a strict time-aware evaluation is contemplated, which is also used in this work. However, in this article, we aim to provide a generic approach to the early detection of cyberbullying that could potentially be applied to any social network.

## 3. Methods

The main purpose of this work is to provide a general perspective for the early detection of cyberbullying on social media. For this reason, we focused our research on the common ground provided by different social media: user comments.

For all experiments, we considered a 5-fold cross-validation scheme to assess the performance and robustness of the models. For each model configuration, each experiment was repeated 5 times by drawing new training and test splits, and results were averaged over 5 rounds. Significance tests were performed using the Welch two-sample *t*-test, and we considered it statistically significant if *p*-value is below 0.01.

### 3.1. Problem Definition

This section provides a formal definition of the cyberbullying early detection problem on social media sessions.

We define $S=\{s_{1},s_{2},\ldots ,s_{n}\}$ as the set of media sessions that may suffer from cyberbullying, where |S|=n denotes the total number of sessions. Each social media session s∈S is composed of a sequence of posts, denoted as $P_{s}$, along with a binary indicator $l_{s}$ that specifies if the session is considered cyberbullying ($l_{s}=true$) or not ($l_{s}=false$).

For each social media session, the sequence of posts increases throughout time, and is denoted as:

(1) $$P_{s}=\left(<P_{1}^{s},t_{1}^{s}>,<P_{2}^{s},t_{2}^{s}>,\ldots ,<P_{m}^{s},t_{m}^{s}>\right)$$

where the tuple $<P_{i}^{s},t_{i}^{s}>,i\in \left[1,m\right]$ represents the *i*-th post for media session *s* and $t_{i}^{s}$ is the timestamp when the post $P_{i}^{s}$ was produced. We define each post, $P_{i}^{s}$, by considering the set of features described in Section 3.2. Each social media session is independent, and therefore, the number of posts for each session, *m*, may be different.

The objective of the cyberbullying early detection problem consists of, given a social media session *s*, detecting whether the session can be considered cyberbullying but examining as few posts from $P_{s}$ as possible. Therefore, the output for early detection models will be binary (i.e., 0 for non-cyberbullying or negative; 1 for cyberbullying or positive), along with the number of posts processed, *k*, to reach the final decision. Note that when processing post *i* and all previous posts $P_{1}^{s},\ldots P_{i-1}^{s}$), the model may produce a nondefinitive decision (i.e., delay), which implies that more posts must be processed to reach a final decision.

### 3.2. Features

Previous works, as seen, for example, earlier in [6], have used specific social media features in their models, but this is precisely what we intend to avoid.

Therefore, we did not take into consideration any number of features regarding users’ followers, media session features such as number of likes or sharing, nor specific media features such as content or emotions extracted from different multimedia (e.g., pictures or videos).

On the contrary, our main and only focus refers to users’ comments on the media provided by the different social networks. Therefore, from the comments for both Instagram and Vine datasets, we considered the following groups of features based on previous works:

- Impolite comments: Following [14,39], we identified profane words for each post, where profane words are obtained from a dictionary. For each comment, we calculated its percentage of profane words, and we defined a comment as rude if it contained at least one profane word. For each session, we computed the percentage of rude comments.
- Sentiment analysis: Following [13], we measured polarity (positive and negative) and subjectivity for each comment. For each session, we calculated average values and the percentage of negative and subjective comments.
- Tf-idf: For each comment, we built a Tf-idf (term frequency–inverse document frequency) representation, following [6]. We performed a grid search for optimal hyperparameters using 10% of the Instagram dataset. Stopwords were removed and unigrams, bigrams, and trigrams were considered. The 100 most frequent n-grams were extracted as features, a minimum document frequency of 2 was required, and terms that appear in more than 90% of the documents were ignored. Features were computed for each individual post and partial session.
- Latent Dirichlet allocation (LDA): Following [6,13], we computed semantic similarity using LDA. Optimal hyperparameters were obtained through grid search: 10 topics and a learning rate of 0.9. Features were computed for each individual post and partial session.

One of the contributions of this work is to explore the impact of Doc2Vec features in cyberbullying early detection. Quoc and Mikolov propose [40] an unsupervised method to provide fixed-length representations from variable-length pieces of texts. We considered that it may be suitable to extract information from social media posts, as it proved to be useful for text feature extraction in topic analysis [41,42].

As in previous features, we performed a grid search to tune hyperparameters: a distributed bag of words algorithm with negative sampling was used for training, the maximum window distance between two terms was set to 5, and 100 features were extracted. Doc2Vec features were extracted for each individual post and for each partial session.

## 4. Experiments

### 4.1. Datasets

In this research, we used two datasets tagged for cyberbullying from two different social networks: Instagram and Vine.

The Instagram social network is a media-based social network mainly focused on images, where users can post and comment on them. The Vine social network is based on a mobile application that allows users to record and edit short videos to publish on their profiles, while other users can watch and comment on these videos.

The Instagram dataset was collected and described in detail by Hosseinmardi et al. [14,39]. The authors started with a sample of 25 thousand Instagram users with public profiles, and from there, a collection of more than 3 million media sessions was extracted. Sessions were limited to those with at least 15 comments, and to achieve a more balanced dataset, only sessions with at least one profane word in their comments were considered.

As a result, 2218 media sessions (with their comments) were labelled for cyberbullying using human contributors. Each contributor received a level of trust based on a series of tests and quizzes. A weighted version of the majority voting method was applied, where a minimum trust of 60% is required to keep the session in the dataset, leaving a total of Instagram sessions.

A similar approach was followed to create the Vine dataset [12,13]. The sample started with more than 650 thousand media sessions, which, after removing those sessions with fewer than 15 comments, was reduced to 436 thousand sessions. Then, the sessions were grouped in terms of the percentage of comments including profane words, and a representative subsample for all groups was considered for labelling, obtaining a total of 969 media sessions. Each one of these sessions was tagged by 5 reviewers, and sessions with a confidence level below 60% were discarded from the dataset, obtaining a final number of 747 Vine sessions.

Table 1 presents the main statistics for both datasets. The Instagram dataset is bigger, both in terms of sessions and comments, while the Vine dataset provides a higher rate of comments per session than Instagram.

**Figure 1:**
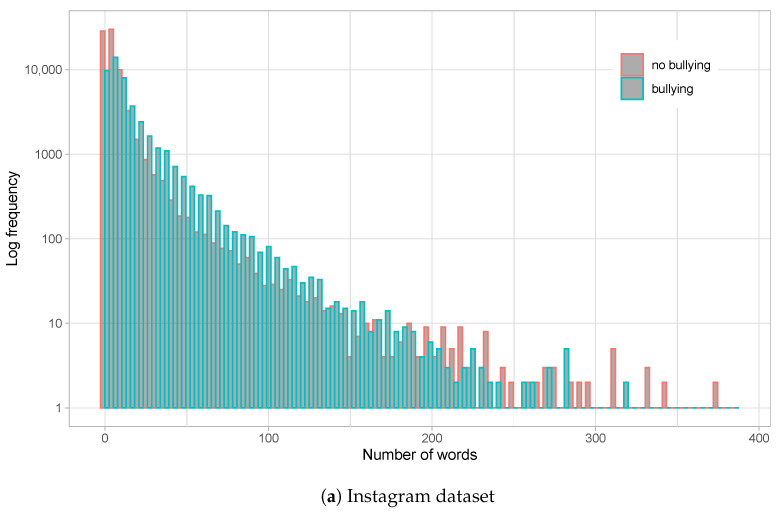

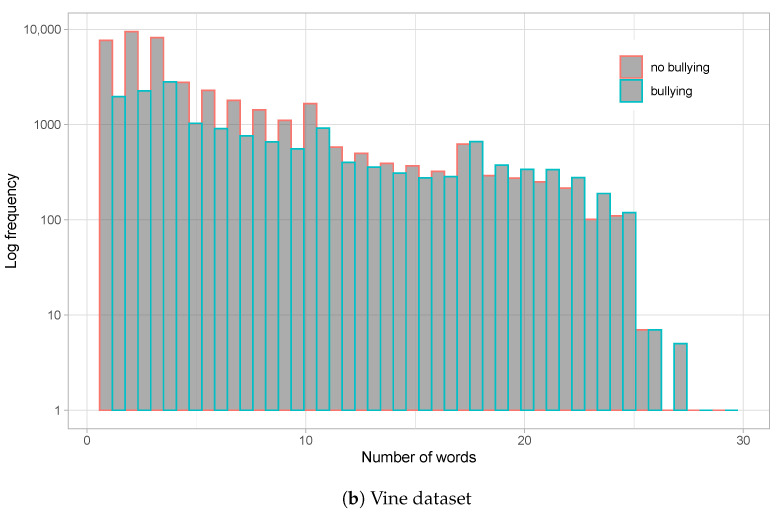
Histograms for number of words on comments for the Instagram dataset (**a**) and Vine dataset (**b**). Logarithmic scale is employed for Y-axis (frequency). Bullying and non-bullying comments are differentiated on each histogram.

We also analysed the main language used on the different social media networks. It is interesting to note that Vine sessions show a higher variability of languages, while most Instagram comments are in English. We consider that this could be motivated by the smaller size of the comments in the Vine dataset (see Figure 1), which points towards the use of more abbreviations and slang.

**Figure 2:**
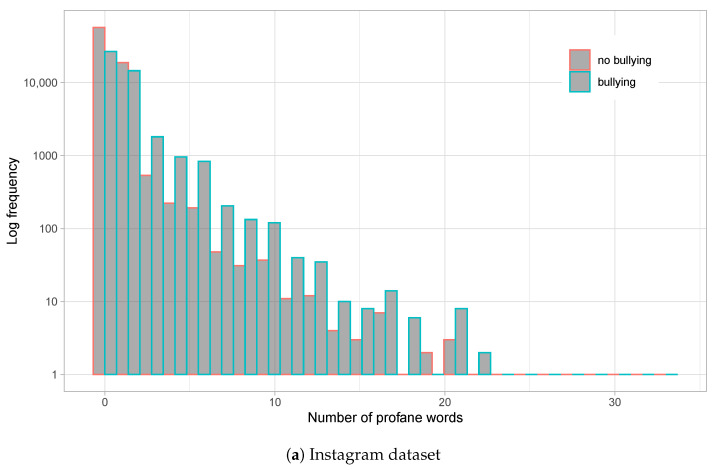

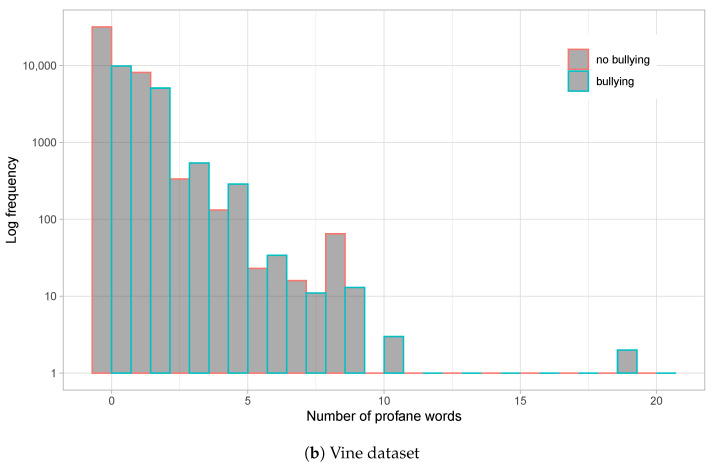
Histograms for profane words usage per comment for the Instagram dataset (**a**) and Vine dataset (**b**). Logarithmic scale is employed for Y-axis (frequency). Bullying and non-bullying comments are differentiated on each histogram.

Figure 1 shows the distribution of the number of words per comment for both datasets, whereas Figure 2 shows the distribution of profane words. From the graphs, we observe that Instagram comments are much longer than those on Vine. With an average of nearly 10 words per comment on Instagram, and a long queue with posts reaching nearly 400 words, a relatively high standard deviation of 17.46 is produced. On the other hand, Vine averages 5.7 words per comment (maximum is around 30 words) and a standard deviation of 5.41, all in all indicating a much smaller post size for this social network. Moreover, Figure 1a shows a slight tendency to use more words on bullying comments on Instagram, which is just minorly present at the tail of the word distribution for the Vine dataset (Figure 1b).

### 4.2. Early Detection Metrics

Timeawareprecision or TaP will be considered as the evaluation metric, as presented in [43] for cybersecurity evaluation purposes.

The timeawareprecision or (TaP) is defined as follows at point *o* for an entity $e_{i}$:

(2) $${\mathrm{TaP}}_{o,\lambda }\left(e_{i},k\right)=\left\{\begin{array}{cc} -1 & \mathrm{if}\;\mathrm{FP}\vee \mathrm{FN}\\ 1 & \mathrm{if}\;\mathrm{TP}\vee \mathrm{TN}\wedge k\leq o\\ 1-pf_{o,\lambda }\left(k\right) & \mathrm{if}\;\mathrm{TP}\vee \mathrm{TN}\wedge k>o\\ 0 & \mathrm{if}\;\mathrm{delay} \end{array}\right.$$

The output of the metric TaP for a set of entities *E* is obtained by the aggregation of individual entities $e_{i}$ by means of an average score, as shown next:

(3) $$TaP_{o,\lambda }\left(E,k\right)=\frac{1}{\left|E\right|}\sum_{e_{i}\in E}TaP_{o,\lambda }\left(e_{i},k\right)$$

where λ is the decay parameter to define how harsh the penalisation applied is, and *k* corresponds with the amount of items being processed at that specific point. Moreover, as previously studied in [43] but not included here for sake of simplicity, an α parameter is included to allow for the balance of cases in different problems.

TaP metric definition, in contrast to other time-aware metrics, is problem-related, and its parameters do not depend on the specific dataset but on the problem being evaluated. This characteristic allows for a more comprehensive approach to the evaluation of early detection models.

### 4.3. Baseline

As early detection baseline models, we consider those described in previous works [6,44,45] that have reported relevant results when evaluated with early detection metrics: fixed, threshold, and dual. To the best of our knowledge, no other previous results using time-aware metrics such as TaP as evaluation metrics for early detection models have been presented.

As introduced, there are few cyberbullying early detection studies employing early detection metrics solely using comment data. On account of the state-of-the-art results, we can cite the following research using the Instagram and Vine datasets. The authors of [27] obtained the best results for the Instagram dataset, using linear regression with an F1 of 0.64±0.03 (precision 0.79±0.03 and recall 0.55±0.03). This is also shown in [29] with an F1 value around 0.65 (precision 0.873±0.04 and recall 0.517±0.05). In the case of the Vine dataset, an F1 of 0.59±0.04 was also reached with linear regression (precision 0.62±0.05 and recall 0.57±0.05). These values, although not directly comparable, can give a reference through the values obtained in [6] for the Vine dataset.

For all our experiments, the following machine learning models were used for fixed, threshold, and dual: AdaBoost (ADA), extra trees (ET), logistic regression (LR), naïve Bayes (NB), and support vector machine (SVM).

- A fixed early detection model consists of a simple adaptation of a standard machine learning model by considering a fixed number of input comments. Meanwhile, a delay is produced until this number is reached; hence, a final prediction will be generated by the model [6]. In our experiments, we consider different values for the number of input comments: 1, 5, 10, 15, and 25. A different performance result is obtained for each fixed point and for each machine learning model. For simplicity, we will denote each model with a subscript at a decision point (e.g., $ADA_{5}$ or $ET_{15}$).
- A threshold early detection model uses a machine learning model, and sets a positive threshold (th+) and a negative threshold (th−) based on the class probability returned by the model to decide when to make a final decision (i.e., cyberbullying or non-cyberbullying) [6]. That is, for a specific media session *s*, after processing post *i* of *s*, $P_{i}$, the model tests whether the class probability is higher or equal to th+ to emit a cyberbullying prediction, and if not, tests whether it is higher or equal to th− to produce a non-cyberbullying prediction. Otherwise, a delay output is generated, and further posts must be processed.
- A dual early detection model relies on two independent machine learning models, with one model (m+) trained to detect cyberbullying cases and another model (m−) trained to detect non-cyberbullying cases. Positive and negative thresholds are also required, but in this case are associated to its own model. Therefore, after processing $P_{i}$ by m+, the class probability must be higher or equal to th+ to be considered cyberbullying, and likewise, when processed by m−, it must be above or equal to th− to be considered non-cyberbullying. In any other case, a delay output is produced.

In this work, we also study the use of multiple instance learning (MIL) for the early detection models. The MIL paradigm assumes that labels are assigned to sets or bags (i.e., media sessions, in our case), but training examples are considered ambiguous: a single object is expected to have multiple alternative instances (i.e., posts, in our case) that describe it, and only some of those may be responsible for the object classification [46]. More specifically, we focus on instance-space methods that obtain bag labels by aggregating individual instance features [47]. In our experiments, we considered the following bag aggregation functions: minimum, maximum, average, median, and average between minimum and maximum values. Therefore, an initial MIL phase is introduced that will provide a new media session representation, and then baseline early detection models can be executed as usual.

### 4.4. Performance Evaluation

The following sections present the results for each of the three sets of experiments performed. First, we study how the inclusion of Doc2Vec features affect all the proposed models. Finally, we incorporate multiple instance learning to fixed early detection and threshold models.

#### 4.4.1. Baseline

In this section, we present the baseline results using the TaP metric, as introduced in [43] for all three models used on the experiments: the fixed model, threshold model, and dual model.

Firstly, in Table 2, it must be noticed that even if the general values differ, the best point of decision for each fixed model is almost the same for all the models and datasets. This can be explained by the fact that the amount of information processed is almost the same for all models and both datasets (i.e., point 10, in eight cases out of ten), which leads to finding the optimal point of decision related to the penalisation applied by the metric. On Instagram, the best baseline performance is obtained with LDA for *naïve Bayes* (at point 10) reaching 0.3755. On the other hand, for the Vine dataset, the best baseline performance combines Tf-Idf and LDA features for *naïve Bayes* (also at point 10), obtaining TaP=0.3794.

Secondly, Table 3 shows the results for the threshold model, where more differences can be seen, as variation in thresholds does not correlate directly with the amount of items processed to make a decision. This contrasts with the results shown previously, but can be explained due to the fact that a higher threshold could mean a lower probability of making the wrong choice, but also could increment the number of items processed to take the final decision. As for the results, focusing on Instagram, the best baseline score is 0.7561, obtained with Tf-idf features, using *naïve Bayes*, and the threshold early detection model parameters are th+=0.5 and th−=0.9. For the Vine dataset, LDA features are used, also using the *naïve Bayes* model, and the threshold parameters are also th+=0.5 and th−=0.9. The performance obtained for the best baseline features in this dataset is TaP=0.399.

Finally, in Table 4, even taking into account the difficulty in the interpretation of the results due to the complexity of the dual model (comprising one model for positive cases and other for negative cases, each one with its own features and thresholds), it can be seen that the same pattern applies, with slight differences in the configuration of the models, as stated for the fixed and threshold baselines. For instance, the same values are obtained for all combinations of negative features in the same models, as is the case of *NB* for both the Instagram and Vine datasets. For these results, different threshold configurations were tested, using low values for the positive threshold and high values for the negative threshold, as in the threshold early detection model. However, for sake of simplicity, in Table 4, we show the results for th+=0.5 and th−=0.9, as this configuration achieved the best results.

In this case, for the Instagram dataset, the best TaP outcome is 0.7561 when just *Tf-idf* are used as positive features, no matter the combination of negative features selected for the *naïve Bayes* model. In the case of the Vine dataset, all combinations of negative features also present the same output, also reaching the highest value at 0.399 for *naïve Bayes* when LDA features are used.

#### 4.4.2. Doc2Vec Features

In this first set of experiments, we evaluated whether Doc2Vec features can significantly improve performance. In all the experiments, sentiment and profane analysis features were included, and different combinations of syntactic and semantic features (i.e., Tf-idf, LDA, and Doc2Vec) were tested.

**Figure 3:**
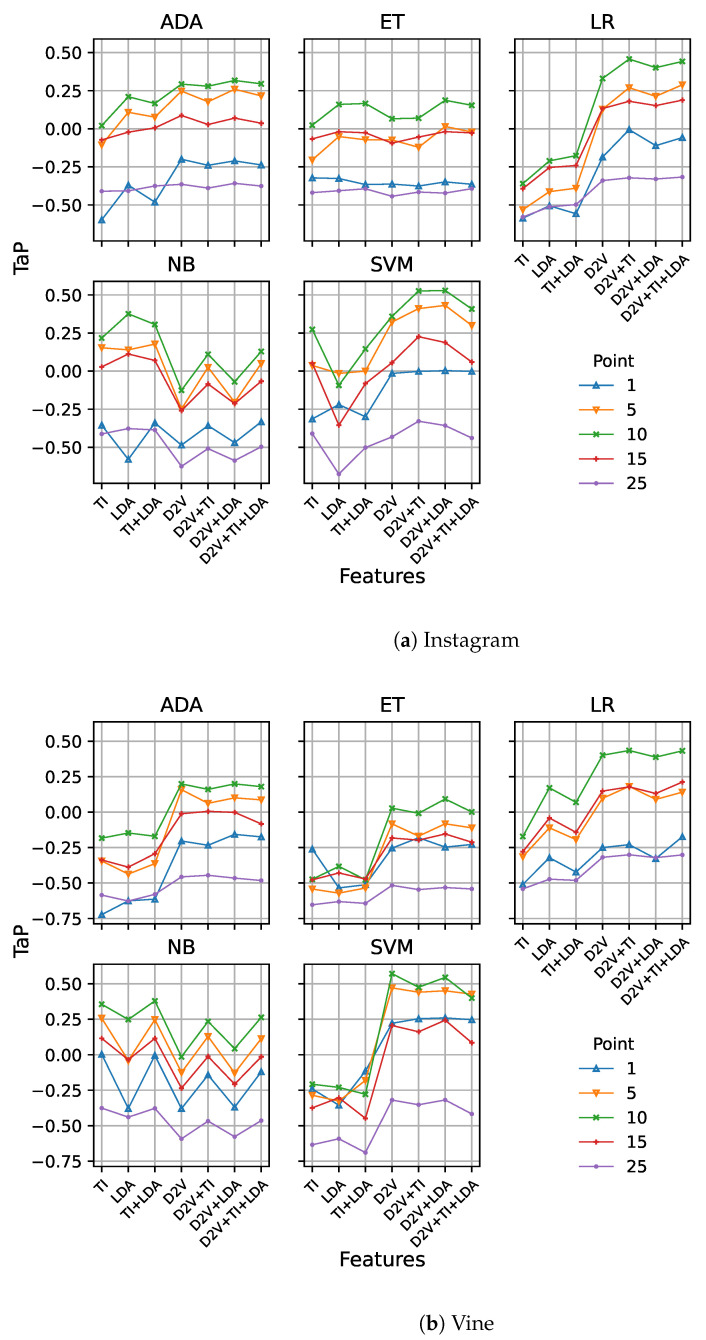
TaP for fixed early detection model at points 1,5,10,15, and 25 using Instagram (**a**) and Vine (**b**) datasets. One graph is presented for each machine learning model: AdaBoost (ADA), extra trees (ET), logistic regression (LR), naïve Bayes (NB), and support vector machine (SVM). Features are represented on the X-axis. Tf-idf (TI), latent Dirichlet allocation (LDA), and Doc2Vec (D2V).

Figure 3a,b show the results for the fixed early detection model at points 1, 5, 10, 15, and 25 for the Instagram and Vine datasets, respectively. The X-axis represents the syntactic and semantic features extracted from the posts. The first three items correspond to standard features (i.e., Tf-idf, LDA, and the combination of both), which constitute our baseline. The remaining items correspond to Doc2Vec and its combination with Tf-idf and LDA features.

From Figure 3a, we observe how the use of Doc2Vec features (stand-alone or in combination) improves performance, especially for the logistic regression and SVM models, and independently from the point when the fixed model makes the decision. This behaviour is clearer on the Vine dataset (Figure 3b), as there is a higher improvement for all machine learning models, except naïve Bayes.

Introducing Doc2Vec features immediately improves performance, but the best-performing model on the Instagram dataset is $SVM_{10}$, combining Doc2Vec with LDA and reaching 0.529 (versus previous 0.3755 value), while for Vine, the use of Doc2Vec reaches up to 0.5716 with SVM (versus 0.3794 previously obtained) also at point 10.

**Figure 4:**
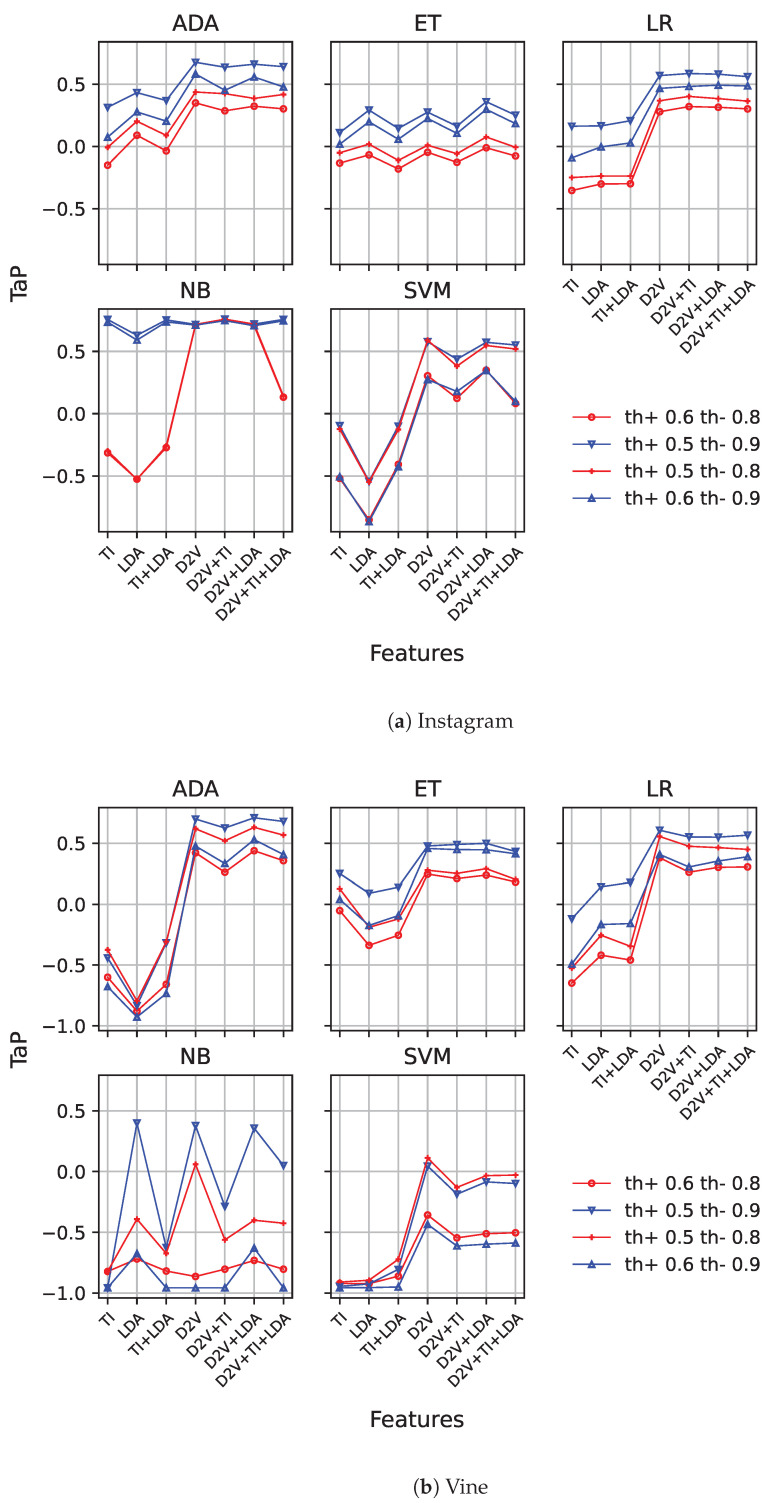
TaP for threshold early detection model using Instagram (**a**) and Vine (**b**) datasets. One graph is presented for each machine learning model. Different values for positive and negative thresholds are represented on each graph. Features are represented on the X-axis.

Following this, we replicated the same experiments for the threshold early detection model. Figure 4a,b show the results for both datasets. Since this model requires positive and negative threshold values, we show different line types for different thresholds. We limited our results to low positive thresholds (i.e., 0.5 and 0.6) and high negative thresholds (i.e., 0.8 and 0.9), since they have provided good results in previous works [6]. The rationale behind these values is clear: to provide an easy-to-surpass threshold for the early detection of cyberbullying cases with little information, while negative cases require a higher degree of confidence.

From the figures, we observe a similar behaviour as in the previous case, with an important performance improvement. By introducing Doc2Vec, in combination with the other features, the performance significantly improves, except in the case of *naïve Bayes* and extra tree, where this difference is lower. With this model, on the Instagram dataset, that improvement is only clearly present for the th+=0.6 and th−=0.8 configuration, while on Vine, a slight increase in the results is present for th+=0.5, with the inclusion of Doc2Vec in some of the combinations of features. In this last case, it obtains worse results without the use of TI features. It must be noticed that in any case, the best results for *naïve Bayes* are higher than the ones achieved with the other models. On Instagram, *naïve Bayes* is the best-performing model, reaching up to 0.7585, using Doc2Vec with Tf-idf features when a low positive threshold is used(th+=0.5), no matter the negative threshold used (th−=0.8 and th+=0.9).

On the other hand, Vine’s best-performing threshold model uses *AdaBoost*, and requires Doc2Vec and LDA features to score 0.7097 (th+=0.5 and th−=0.9), significantly improving the baseline performance.

**Figure 5:**
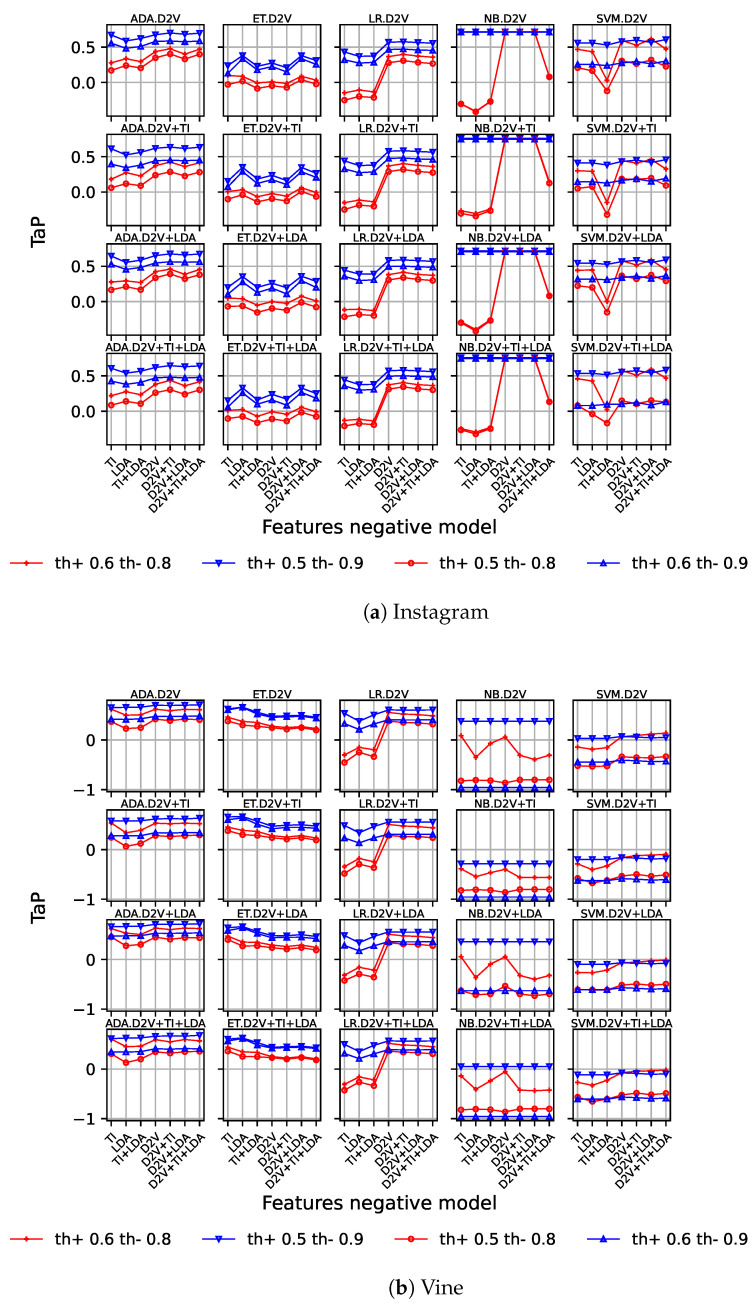
TaP for dual early detection model using Instagram (**a**) and Vine (**b**) datasets. Columns correspond to machine learning models. Rows correspond to positive features. Negative features are represented on the X-axis. Different values for positive and negative thresholds are represented on each graph.

Finally, in this set of experiments, we validated the Doc2Vec behaviour for the dual model, and the results are shown on Figure 5a,b. Each column represents the results for one machine learning model, while rows correspond to positive features. Since the best scores were achieved using Doc2Vec as positive features, we limited the rows to those cases. As in the previous figures, negative features are represented on the X-axis, and different values for positive and negative thresholds are represented on each graph.

Focusing on the Instagram dataset (Figure 5), the best-performing models are AdaBoost and naïve Bayes, and th−=0.9 tends to achieve better results in most cases. The best score is 0.7585, which corresponds to naïve Bayes using Doc2Vec and Tf-idf as positive features for all negative feature combinations with a low positive threshold (th+=0.5).

With respect to the Vine dataset (Figure 5b), we observed a limited impact on all machine learning models for some feature combinations, both positive and negative. Just low negative thresholds (th−=0.8) present a notable variation for logistic regression model. In the rest of the cases, the performance remains quite stable. AdaBoost and extra trees achieve good results in general and present a consistent behaviour, independently of positive and negative thresholds. In fact, the best score (0.7167) is obtained by AdaBoost, using all features as negative and Doc2Vec and LDA as positive, with th+=0.5 and th−=0.9. This result significantly improves the dual model baseline performance for the Vine dataset.

Table 5 summarises the main results for this first set of experiments, comprising the inclusion of Doc2Vec features. We observe that its incorporation significantly improves the results obtained by the early detection models for both datasets. Interestingly, the performance improvement is more important on the Vine dataset. We consider that this may be motivated by the smaller post sizes (in terms of word count) and the limitations of Tf-idf and LDA to extract valid features, while Doc2Vec is able to better capture the information available.

Regarding the machine learning models, naïve Bayes for the Instagram dataset and AdaBoost for Vine provide the best results. Another interesting point is the fact that the threshold and dual models significantly improve performance over the fixed model for all cases, except for the threshold model on the Vine dataset. However, there is no significant statistical difference in performance between them. In the remaining experiments, we focus on the threshold early detection model, since it requires a simpler configuration, and the performance results can be considered equivalent to the dual model.

#### 4.4.3. Multiple Instance Learning

In the final set of experiments, we study whether the use of MIL has a positive impact on the early detection of cyberbullying measured with TaP early detection metric. With this aim, we incorporated MIL to the fixed early detection model by adding a bag representation of the posts processed. Those bags of labels were generated on a previous step to ML model training. This addresses the problem of ambiguity generated by single objects while entities with multiple items are supposed to have multiple alternative instances. The features from the posts in the bag are aggregated using the following functions: minimum, maximum, average between maximum and minimum, arithmetic mean, and median.

**Figure 6:**
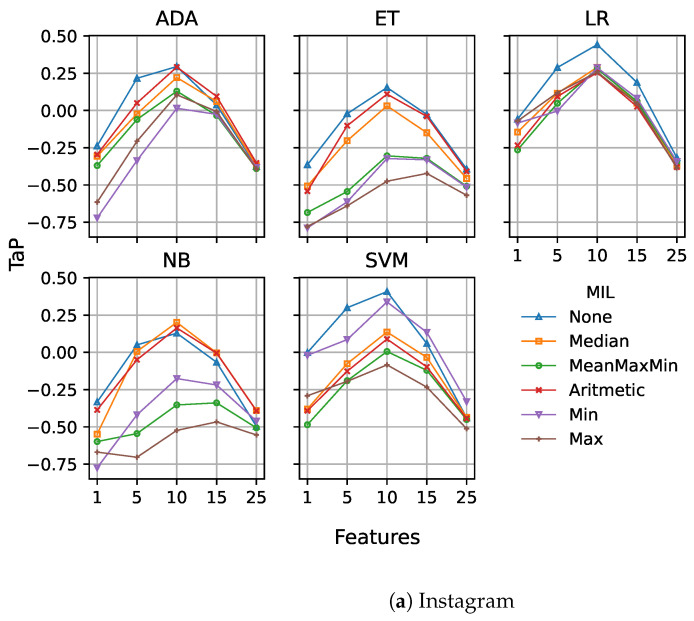

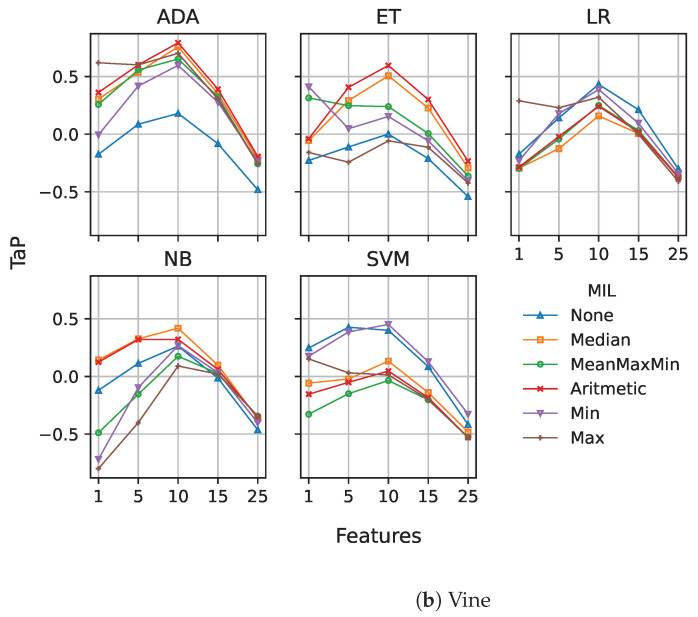
TaP for fixed early detection model based using MIL for Instagram (**a**) and Vine (**b**) datasets. One graph is presented for each machine learning model. The X-axis represents the points where the fixed model produces its decision. Different aggregation functions are represented on each graph: no MIL (None), minimum (Min), maximum (Max), average maximum and minimum (MeanMaxMin), arithmetic mean (Arithmetic), and median (Median).

To help the comparison, we included an aggregation function, denoted as None, that represents a fixed early detection model without MIL. In all our experiments, we ran models with the combination of all features defined: profane words, sentiment analysis, Tf-idf, LDA and Doc2Vec. Figure 6a,b show the results obtained for the fixed model.

Regarding the Instagram dataset (Figure 6a), the use of MIL, in many cases, reduces the performance of the model. There are some slight improvements for all models (except logistic regression) at the higher points. In fact, the best score using MIL is obtained by SVM at point 10 using the minimum as aggregation function, and corresponds to 0.3384. However, there is no statistical difference in comparison with the fixed model without MIL, and it is significantly worse than the threshold and dual models.

However, when considering the results for the Vine dataset (Figure 6b), there is a significant change, especially for the AdaBoost and extra trees models, with MIL models providing major improvements. The best score in this case is 0.7926, achieved by AdaBoost at point 10 using the arithmetic mean, which is significantly better than the results without MIL for the fixed, threshold, and dual models.

We consider that this important performance improvement by using multiple instance learning for the Vine dataset is directly related to the reduced post sizes on this social network and the lower use of standard English. On average, each post is composed of about 5 words, which provides limited information to be extracted by the different syntactic and semantic features for each of them. However, the use of a simple aggregation function (e.g., average) for a number of posts allows for better combination of the information from the different posts than the concatenation of the posts themselves. On the other hand, Instagram posts are larger and mostly in English, and therefore, the features extracted provide sufficient information from each post, making the aggregation less relevant.

We also conducted experiments testing different sampling alternatives for the training set, instead of using all post sessions. In particular, we analysed using the first 10 posts, the last 10 posts, or 10 random posts, with no relevant improvements obtained for the fixed early detection model using MIL.

**Figure 7:**
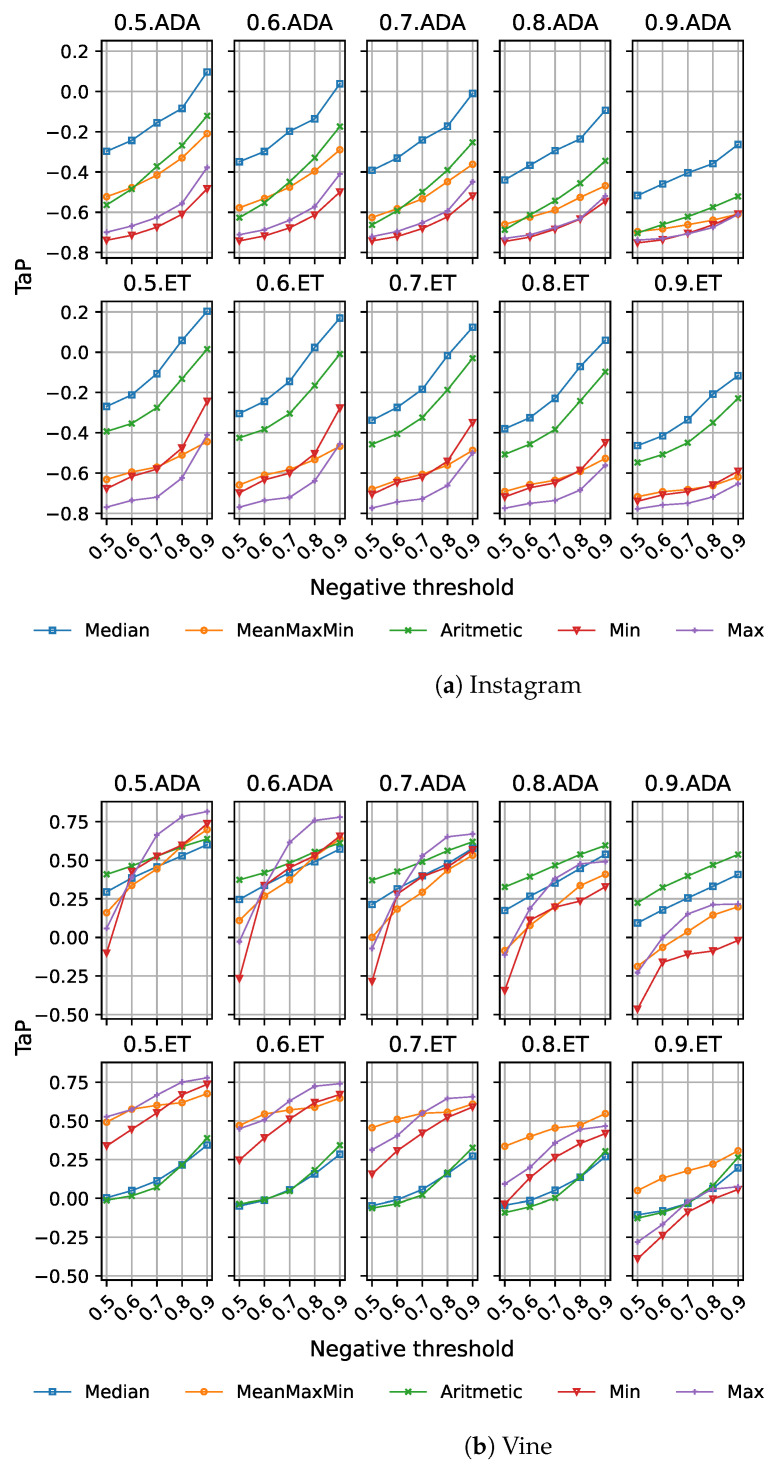
TaP for threshold early detection model based using MIL for Instagram (**a**) and Vine (**b**) datasets. Rows correspond to machine learning models: ADA and ET. Columns correspond to positive thresholds. The X-axis represents negative thresholds. Different aggregation functions are represented on each graph: minimum (Min), maximum (Max), average maximum and minimum (MeanMaxMin), arithmetic mean (Arithmetic), and median (Median).

We also explored the impact of MIL on the threshold early detection model performance. The experiments were limited to the AdaBoost and extra trees models, since they provided the highest improvements on our previous experiments, and again, we used all features in our models. In this case, due to the important change on the features introduced by MIL, we considered the whole range for positive and negative thresholds: 0.5, 0.6, 0.7, 0.8, and 0.9. Figure 7a,b present the results for these experiments.

The behaviour on the Instagram dataset (Figure 7a), as expected, is below the results achieved by the baselines provided in Section 4.4.2 for the fixed, threshold, and dual models (being significantly worse for the last two). Increasing the negative threshold improves performance, and the median aggregation function is consistently providing the best results in all cases, but there is no significant improvement in TaP results for this dataset.

Regarding the Vine dataset (Figure 7b), we observe how AdaBoost provides a better performance than extra trees. As in the previous case, increasing the negative threshold improves performance, while for the positive threshold, the best values are obtained with 0.5 and 0.6. This is a meaningful change with respect to the standard threshold model, motivated by the aggregation of information from multiple posts, which produces an increase in the class probability for the cyberbullying cases detected. From the aggregation functions, the maximum concentrates the higher scores.

In fact, the best score of 0.8149 is obtained by AdaBoost using the maximum and th+=0.5 and th−=0.9. Again, this model significantly improves performance over the best fixed, threshold, and dual models in Section 4.4.2, although there is no statistical difference with respect to the fixed model from our previous experiments.

## 5. Results and Discussion

All experiments were performed with two different social network datasets and evaluated under an early detection metric. This allowed for a proper evaluation from the early detection’s point of view, as the delay is taken into account in the evaluation. In addition, the experiments were performed on datasets of different natures, as can be seen in the outcome of the analysis. They were performed solely with textual information, disregarding platform-dependent information. This draws attention to the syntactic and semantic features extracted from posts.

Considering as a starting point the early detection models and features from our previous work [6], we have proven how Doc2Vec features significantly improve performance for all early detection models, with threshold and dual performing similarly. The best TaP scores achieved for the Instagram and Vine datasets were, respectively, 0.7585 and 0.7167, improving the best respective baseline models in both cases.

We have also shown how the introduction of multiple instance learning in early detection models has an important positive effect on the Vine dataset results, motivated by the smaller post sizes and lower use of standard English language, while there is no significant improvement on Instagram. The threshold model is able to reach a score of 0.8149 with the AdaBoost model for the Vine dataset.

In particular, in the Doc2Vec features section (Section 4.4.2), we assess the performance improvement for all early detection models. We find that it provides an important performance enhancement for threshold and dual models. As there is no significant statistical difference between them, the latter would be preferable due to its simpler configuration. Finally, in section MIL Section 4.4.3, we incorporate multiple instance learning to the fixed and threshold models. It does not provide a better performance for the Instagram dataset, but it improves the TaP score for the Vine dataset. This is related to the reduced sizes of the posts in the dataset and the lower use of English.

## 6. Conclusions and Future Work

In this work, we have explored the cyberbullying early detection problem on social networks from a generic perspective, focusing only on users’ comments.

To summarise, the inclusion of Doc2Vec features improves all early detection models’ performance, in particular threshold and dual. Moreover, multiple instance learning has shown better results for the Vine dataset, with smaller post sizes and lower use of the English language.

In future works, we expect to continue exploring multiple instance learning by using more advanced instance-space MIL models, such as expectation–maximisation diverse density (EMDD), and also considering bag-space and embedded-space models, such as normalised set kernel (NST-SVM) or multiple instance learning via embedded instance selection (MILES). In that sense, different combinations of these methods after grouping by post types and subject matter could be studied. Related to this, different kinds of filters could be applied in order to obtain specific characteristics of those interactions.

We also consider that the early detection problem can be applied in multiple and different environments, and hence, we expect to provide a generic approach to this problem with a suitable evaluation methodology and metrics.

## Figures and Tables

**Table 1:** Statistics for Instagram and Vine datasets.

	Instagram			Vine		
	Cyberbullying	Normal	Total	Cyberbullying	Normal	Total
Media sessions	585	1369	1954	190	557	747
	29.94%	70.06%	100%	25.44%	74.56%	100%
Comments	45,372	76,862	122,234	15,810	40,389	56,199
	37.12%	62.88%	100%	28.13%	71.87%	100%
Comments/session	77.56	56.14	62.56	83.21	72.51	75.23
Words/comment	14.00	7.13	9.68	7.64	4.96	5.72
English	74.16%	73.22%	73.74%	47.62%	34.43%	38.69%

**Table 2:** TaP for fixed model using only baseline features: Tf-idf (TI) and latent Dirichlet allocation (LDA). The first column corresponds to features and the second column to point of decision. Results are grouped by datasets (i.e., Instagram and Vine), and each column corresponds to one machine learning model. Best score for each model is underlined and best score for each dataset is highlighted in bold.

Features		Instagram					Vine				
Features	Points	ADA	ET	LR	NB	SVM	ADA	ET	LR	NB	SVM
TI	1	−0.5975	−0.3222	−0.5864	−0.3545	−0.3138	−0.7228	−0.2609	−0.5100	0.0036	−0.2414
	5	−0.1060	−0.2044	−0.5313	0.1531	0.0370	−0.3444	−0.5424	−0.3139	0.2577	−0.2854
	10	0.0204	0.0236	−0.3601	0.2172	0.2734	−0.1836	−0.4737	−0.1720	0.3561	−0.2080
	15	−0.0732	−0.0664	−0.3933	0.0276	0.0520	−0.3373	−0.4774	−0.2795	0.1156	−0.3744
	25	−0.4097	−0.4191	−0.5794	−0.4122	−0.4101	−0.5851	−0.6533	−0.5414	−0.3761	−0.6345
LDA	1	−0.3700	−0.3265	−0.5055	−0.5793	−0.2195	−0.6255	−0.5342	−0.3215	−0.3793	−0.3555
	5	0.1080	−0.0512	−0.4127	0.1395	−0.0151	−0.4367	−0.5712	−0.1107	−0.0412	−0.3269
	10	0.2097	0.1597	−0.2108	**0.3755**	−0.0932	−0.1472	−0.3829	0.1708	0.2485	−0.2300
	15	−0.0223	−0.0195	−0.2541	0.1123	−0.3550	−0.3880	−0.4290	−0.0415	−0.0352	−0.3031
	25	−0.4077	−0.4067	−0.5121	−0.3771	−0.6757	−0.6270	−0.6308	−0.4729	−0.4396	−0.5923
TI+LDA	1	−0.4815	−0.3662	−0.5575	−0.3397	−0.2993	−0.6135	−0.5122	−0.4226	−0.0046	−0.1147
	5	0.0761	−0.0723	−0.3900	0.1774	0.0000	−0.3632	−0.5350	−0.1935	0.2480	−0.1803
	10	0.1659	0.1658	−0.1764	0.3060	0.1453	−0.1705	−0.4771	0.0688	**0.3794**	−0.2787
	15	0.0056	−0.0265	−0.2412	0.0701	−0.0817	−0.2931	−0.4716	−0.1426	0.1153	−0.4486
	25	−0.3754	−0.3938	−0.4976	−0.3861	−0.5017	−0.5796	−0.6435	−0.4811	−0.3780	−0.6897

**Table 3:** TaP for threshold model using only baseline features: Tf-idf (TI) and latent Dirichlet allocation (LDA). The first column corresponds to features, the second column to positive threshold, and the third to negative threshold. Results are grouped by datasets (i.e., Instagram and Vine), and each column corresponds to one machine learning model. Best score for each model is underlined and best score for each dataset is highlighted in bold.

			Instagram					Vine				
Features	Th+	Th−	ADA	ET	LR	NB	SVM	ADA	ET	LR	NB	SVM
TI	0.5	0.8	−0.0077	−0.0509	−0.2500	−0.2986	−0.1241	−0.3751	0.1267	−0.5252	−0.8225	−0.9098
	0.5	0.9	0.3136	0.1106	0.1623	**0.7561**	−0.0970	−0.4404	0.2510	−0.1199	−0.9563	−0.9449
	0.6	0.8	−0.1509	−0.1339	−0.3540	−0.3142	−0.5194	−0.6003	−0.0520	−0.6480	−0.8225	−0.9213
	0.6	0.9	0.0743	0.0176	−0.0918	0.7346	−0.5075	−0.6779	0.0399	−0.4913	−0.9563	−0.9563
LDA	0.5	0.8	0.2022	0.0182	−0.2374	−0.5271	−0.5481	−0.7947	−0.1892	−0.2547	−0.3915	−0.8941
	0.5	0.9	0.4318	0.2915	0.1651	0.6294	−0.5435	−0.8331	0.0901	0.1440	**0.3990**	−0.9247
	0.6	0.8	0.0896	−0.0678	−0.3015	−0.5257	−0.8515	−0.8793	−0.3377	−0.4189	−0.7190	−0.9224
	0.6	0.9	0.2770	0.1976	−0.0034	0.5910	−0.8672	−0.9274	−0.1740	−0.1664	−0.6755	−0.9550
TI+LDA	0.5	0.8	0.0884	−0.1117	−0.2385	−0.2605	−0.1272	−0.3082	−0.1204	−0.3457	−0.6740	−0.7219
	0.5	0.9	0.3686	0.1445	0.2062	0.7529	−0.0996	−0.3170	0.1402	0.1786	−0.6250	−0.8050
	0.6	0.8	−0.0354	−0.1807	−0.2996	−0.2721	−0.4075	−0.6584	−0.2547	−0.4584	−0.8186	−0.8607
	0.6	0.9	0.2031	0.0575	0.0280	0.7368	−0.4260	−0.7341	−0.0931	−0.1586	−0.9563	−0.9498

**Table 4:** TaP for dual model using only baseline features: Tf-idf (TI) and latent Dirichlet allocation (LDA). The first column corresponds to positive features and the second column to negative ones. Results are grouped by datasets (i.e., Instagram and Vine), and each column corresponds to one machine learning model. Best score for each model is underlined and best score for each dataset is highlighted in bold.

Features		Instagram					Vine				
Positive	Negative	ADA	ET	LR	NB	SVM	ADA	ET	LR	NB	SVM
TI	TI	0.3136	0.1106	0.1623	**0.7561**	−0.0970	−0.4404	0.2510	−0.1199	−0.9563	−0.9449
	LDA	0.2609	0.3230	0.1524	**0.7561**	−0.1006	−0.4449	0.3004	−0.1630	−0.9563	−0.9438
	TI+LDA	0.2936	0.1735	0.1716	**0.7561**	−0.0984	−0.4410	0.2526	−0.1097	−0.9563	−0.9449
LDA	TI	0.4710	0.1283	0.1791	0.6294	−0.5398	−0.8287	0.0298	0.1981	**0.3990**	−0.9258
	LDA	0.4318	0.2915	0.1651	0.6294	−0.5435	−0.8331	0.0901	0.1440	**0.3990**	−0.9247
	TI+LDA	0.4464	0.1797	0.1759	0.6294	−0.5413	−0.8293	−0.0004	0.2046	**0.3990**	−0.9258
TI+LDA	TI	0.3858	0.0882	0.2216	0.7529	−0.0981	−0.3165	0.1549	0.1741	−0.6250	−0.8050
	LDA	0.3472	0.2646	0.1976	0.7529	−0.1018	−0.3209	0.2302	0.1034	−0.6250	−0.8039
	TI+LDA	0.3472	0.2646	0.1976	0.7529	−0.1018	−0.3209	0.2302	0.1034	−0.6250	−0.8039

**Table 5:** TaP summary results for early detection models.

	Instagram			Vine		
Model	Baseline	Doc2Vec	%	Baseline	Doc2Vec	%
Fixed	0.3755 (NB)	0.5290 (SVM)	40.8%	0.3794 (NB)	0.5716 (SMV)	50.6%
Threshold	0.7561 (NB)	0.7585 (NB)	0.3%	0.3990 (NB)	0.7097 (ADA)	77.8%
Dual	0.7561 (NB)	0.7585 (NB)	0.3%	0.3990 (NB)	0.7167 (ADA)	79.6%

## Abbreviations

The following abbreviations are used in this manuscript:

MDPI	Multidisciplinary Digital Publishing Institute
LDA	Latent Dirichlet Allocation
MIL	Multiple Instance Learning
SVM	Support Vector Machines
Tf-idf	Term Frequency–Inverse Document Frequency
ET	Extra Trees
LR	Logistic Regression
NB	Naïve Bayes
D2V	Doc to Vec
EMDD	Expectation–Maximisation Diverse Density
NST-SVM	Normalized Set Kernel Support Vector Machines
MILES	Multiple Instance Learning via Embedded Instance Selection
